# Effect of nickel doping at the Mn site on the structural, magnetic and magnetocaloric properties of the Pr_0.55_Sr_0.45_Mn_1−*x*_Ni_*x*_O_3_ (*x* = 0.0, 0.05, 0.1 and 0.15) manganites

**DOI:** 10.1039/d5ra09742h

**Published:** 2026-02-17

**Authors:** A. Selmi, H. Ayed, Malek Gassoumi, W. Cheikhrouhou-Koubaa, E. K. Hlil, Abdelaziz Bouazizi

**Affiliations:** a Equipe Dispositifs Electroniques Organiques et Photovoltaïque Moléculaire, Laboratoire de La Matière Condensée et des Nanosciences, Faculté des Sciences de Monastir Avenue de L'environnement 5019 Monastir Tunisia bohmids@gmail.com; b LT2S Lab, Digital Research Centre of Sfax, Sfax Technopark BP 275 3021 Sakiet-Ezzit Tunisia; c Univ. Grenoble Alpes, CNRS, Grenoble INP, Institut Néel 38000 Grenoble France

## Abstract

The structural X-ray diffraction (XRD), magnetic, critical behavior, and magnetocaloric characteristics of polycrystalline Pr_0.55_Sr_0.45_Mn_1−*x*_Ni_*x*_O_3_ manganites with 0%, 5%, 10% and 15% of Ni doping were investigated in the current work. A solid-state reaction using the stoichiometric powder mixtures of binary oxides at high temperatures was employed for the synthesis of all the samples. The Rietveld refinement of the XRD patterns indicate that the compounds crystallize in an orthorhombic structure with the *Pnma* space group. Temperature- and field-dependent magnetization experiments show a second-order ferromagnetic (FM) to paramagnetic (PM) phase transition in all the samples. However, when the Ni (%, *x*) content increases from 0.00 to 0.15, the Curie temperature (*T*_C_) decreases from 300 K to 180 K. For all the compounds, Arrott plots reveal a second-order phase transition. Using various methods, data from static magnetization measurements at the FM–PM phase transition is used to analyze the critical parameters. Using the isothermal magnetization data around *T*_C_, the magnetocaloric effect (MCE) in terms of maximum entropy change (−Δ*S*^max^_M_) and relative cooling power (RCP) are determined using Maxwell thermodynamic relations. In a *µ*_0_*H* = 5 T magnetic field shift, the highest values of magnetic entropy changes (−Δ*S*^max^_M_) are calculated to be 3.8 J kg^−1^ K^−1^, 1.71 J kg^−1^ K^−1^, 1.62 J kg^−1^ K^−1^, and 1.29 J kg^−1^ K^−1^ for 0%, 5%, 10% and 15% of Ni, respectively. The maximum relative cooling power (RCP) values for *x* = 0.0, *x* = 0.05, *x* = 0.1, and *x* = 0.15 at *µ*_0_*H* = 5 T are 247 J kg^−1^, 216 J kg^−1^, 211 J kg^−1^, and 215.94 J kg^−1^, respectively. The RCP of compound (0% of nickel) = 254.1 J kg^−1^ is 56% of the RCP value of Gd gadolinium metal. These important features make these new materials extremely promising for magnetic refrigeration from a technical standpoint.

## Abstract 2

The structural XRD, magnetic, and magnetocaloric properties of a series of Pr_0.55_Sr_0.45_Mn_1−*x*_Ni_*x*_O_3_ manganite compounds, where *x* = 0.0, 0.05, 0.1 and 0.15, are discussed in detail in this work.

## Graphical abstract

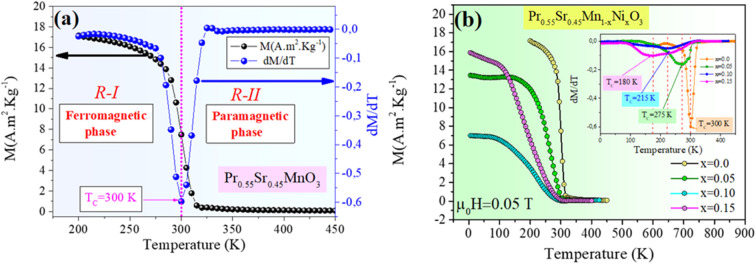

## 1. Introduction

Compared with traditional gas-compression refrigeration, magnetic refrigeration based on the magnetocaloric effect (MCE) has garnered significant attention in recent years because of its superior energy efficiency and ecologically friendly technology.^1,2^

The magnetocaloric effect (MCE) describes how the refrigerant temperature changes in response to variations in the external magnetic field. This phenomenon results from a shift in the magnetic moment alignment when a magnetic field is applied, which is a natural property of magnetic materials. For a magnetic material with a large magnetocaloric effect (MCE), there will be a sharp ferromagnetic–paramagnetic (FM–PM) transition at the Curie temperature, *T*_C_, along with a substantial spontaneous magnetization. Due to its large effective Bohr magneton and high Curie temperature −Δ*S*^max^_M_ = 10.2 J Kg^−1^ K^−1^ at *µ*_0_Δ*H* = 5 T, *T*_C_ = 294 K,^3^ gadolinium Gd is considered one of the most promising materials for magnetocaloric refrigeration.

Nevertheless, the high cost of Gd limits its practical use. As a result, one of the primary research topics in this discipline is the search for new working substances with a large magnetocaloric effect (MCE) and low cost. Currently, besides a few potential materials such as MnAs_1−*x*_Sb_*x*_,^4^ Gd_5_(Si_2_Ge_2_)_4_,^5^ Tb_0.4_Gd_0.6_Al_2_ (ref. 6) and Mn_1.1_Fe_0.9_(P_0.8_Ge_0.2_),^7^ perovskite manganites with the general formula of R_1−*x*_A_*x*_MnO_3_, where R = a rare earth element (Pr^3+^, La^3+^, and Sm^3+^) and A = an alkaline earth metal (Bi^2+^, Sr^2+^, Ba^2+^, and Ca^2+^), have attracted a lot of attention in the last three decades owing to their distinctive physical properties, such as phase separation, charge and orbital ordering, metal-to-insulator transition, and magnetocaloric effect.^8,9^ In this study, we focus on a family of perovskite compounds.

The manganite-family perovskites with various doping atoms have exceptionally high relative cooling power (RCP), making them suitable for use as clean and low-cost refrigeration sources.^10^ Perovskite manganite compounds have recently been used in a number of applications, including magnetic refrigeration systems,^10^ magnetoresistance,^11,12^ and high-efficiency photovoltaic solar cells.^13^ The so-called “magnetic refrigeration” is based on the magnetocaloric effect (MCE), which was discovered by Warburg in 1881 (ref. 14) and has since been thoroughly investigated.

The critical nature of manganites at the PM–FM phase transition has been the subject of numerous studies.^15–19^ There are a number of ways to improve the physical properties of R_1−*x*_A_*x*_MnO_3_ perovskite manganites, such as substituting a divalent element A^2+^ for the trivalent rare earth ions R^3+^ in the perovskite structure. The partial substitution of R^3+^ results in a mixed valence of manganese (Mn^3+^ and Mn^4+^). The appearance of ferromagnetic (FM) order of the spins of the Mn ions, which causes an electron, *e*_g_, to become itinerant and hop from an Mn cation *via* an oxygen anion to another manganese with an empty *e*_g_ band, is the basis for the changes in the physical properties of these perovskite manganites. One of the most important characteristics of manganites is the strong correlation among their structural, electrical, and magnetic properties. This relationship can be explained by the double exchange (DE) mechanism, which Zener initially predicted in 1951.^20^ Various recent investigations^21–23^ on manganites have shown that the substitution of rare earths has an indirect influence on their conduction mechanism, changing the bandwidth and angle of the connection between nearby manganese ions. An alternative approach involves examining the effects of Mn doping through different elements, as it is indisputable that Mn ions contribute substantially to the mechanism of double-exchange (DE) interactions. Several studies and projects^24–28^ have been carried out recently to comprehend the consequences of replacing manganese at the B-site with a transition element. It has been demonstrated that the addition of a transition metal with an electronic configuration distinct from Mn should considerably change the configurations of Mn and the substituent elements. As a result, the conduction mechanism is directly impacted by the replacement of Mn, making it possible to modify the physical characteristics of manganite systems more effectively.

Moreover, the resistivity increases and the Curie temperature (*T*_C_) and magnetism decrease when trivalent and tetravalent elements are substituted for Mn. However, the exact effect is primarily determined by the type of substituent. One such element that influences the structural and magnetic properties of manganites is nickel (Ni). Doping of Mn by magnetic Ni has been reported in various studies.^29–31^ These studies demonstrate that the addition of nickel (Ni) to both systems gives similar results, which include a decrease in the temperature of the magnetic transition from the ferromagnetic (FM) state to the paramagnetic (PM) state as the rate of substitution *x* increases.

In addition, the strong electron–phonon interaction known as the Jahn–Teller effect,^32^ double-exchange interaction (DE)-connected Mn^3+^/Mn^4+^ ions,^33^ an *d* substitution of Mn sites by other elements^34,35^ all have an effect on the <*θ*_Mn–O–Mn_> networks. These factors explain the features of the magnetocaloric effect.

The consequence of doping with Ni is the replacement of Mn atoms by Ni^2+^ ions and modification of the balance between Mn^3+^–O–Mn^4+^ double-exchange and Ni^2+^–O–Mn superexchange interactions.^36,37^ Alternatively, the charge transfer process, which is mediated by the oxygen 2p orbitals, influences the local spin configurations^38,39^ by promoting significant hybridization between the Ni 3d and Mn 3d states. Furthermore, experimental investigations employing XRD and other techniques have shown that Ni doping alters the Jahn–Teller distortions and lattice parameters, which in turn affect the magnetic and magnetocaloric properties.^40^ These charge and spin transfer pathways are further supported by theoretical insights from DFT + U calculations, which emphasize the competition between double-exchange and superexchange interactions,^41^ where Ni doping lowers the Jahn–Teller distortions by replacing the Jahn–Teller-active Mn^3+^ ions. This makes the local environment more symmetric and modifies the lattice properties.^42^

Pr_1−*x*_Sr_*x*_MnO_3_ is one of the most studied manganites; it undergoes a paramagnetic metal to ferromagnetic metal transition at *T*_C_ ≈ 301 K and displays a −Δ*S*^max^_M_ of about 3 J kg^−1^ K^−1^ under an applied magnetic field of *µ*_0_*H* = 5 T.^43^ The double exchange (DE) coupling between Mn^3+^ and Mn^4+^ ions *via* oxygen can decrease as a result of the Ni doping at the Mn site, altering the Mn^3+^/Mn^4+^ ratio.^44^ This type of doping lowers *T*_C_ by reducing the number of mobile electrons. The magnetic phase transition of these manganites is a significant feature. Analysis of their structural and magnetic characteristics will be beneficial in comprehending the impact of Ni on their magnetocaloric properties and provide a comprehensive understanding of the phase transition from FM to PM.

The structural XRD, magnetic, and magnetocaloric properties of a series of Pr_0.55_Sr_0.45_Mn_1−*x*_Ni_*x*_O_3_ manganite compounds, where *x* = 0.0, 0.05, 0.1 and 0.15 are discussed in detail in this work. The Rietveld technique for analysing the diffraction patterns of rayon presents a way to precisely determine the structural properties of materials. To do this, we calculated the relative cooling power (RCP), an essential performance metric, and carefully investigated the magnetocaloric effect.

## 2. Experimental procedure

Pr_0.55_Sr_0.45_Mn_1−*x*_Ni_*x*_O_3_ where *x* = 0.0, 0.05, 0.1 and 0.15 was synthesized at high temperature using the conventional solid-state reaction approach by combining Pr_6_O_11_, SrCO_3_, MnO_2_ and NiO in the appropriate ratios to achieve 99.9% purity. The first elements were well mixed in an agate mortar. To improve crystallization with interim regrinding and repelling, the resulting powders were then compressed into pellets (approximately 1 mm thick) and sintered at 900 °C and 1100 °C for 24 h and 1200 °C and 1300 °C for 12 h for each cycle.^45,46^ Powder X-ray diffraction (XRD) was used for evaluating the phase purity, homogeneity, and cell dimensions at room temperature using a “PAnalytical-X-pert-Pro” diffractometer with Cu-Kα radiation (*λ* = 1.5406 Å).

The FullProf software was used for the structural analysis using the standard Rietveld method.^47–49^ Magnetization was detected using a vibrating sample magnetometer (VSM) at temperatures between 20 and 400 K and magnetic applied fields up to *µ*_0_*H* = 5 T. The MCE results were deduced from the magnetization measurements at applied magnetic fields up to 5 T at various temperatures.

## 3. Results and discussion

### 3.1 Structural properties: X-ray diffraction (XRD) results

**Fig. 1:**
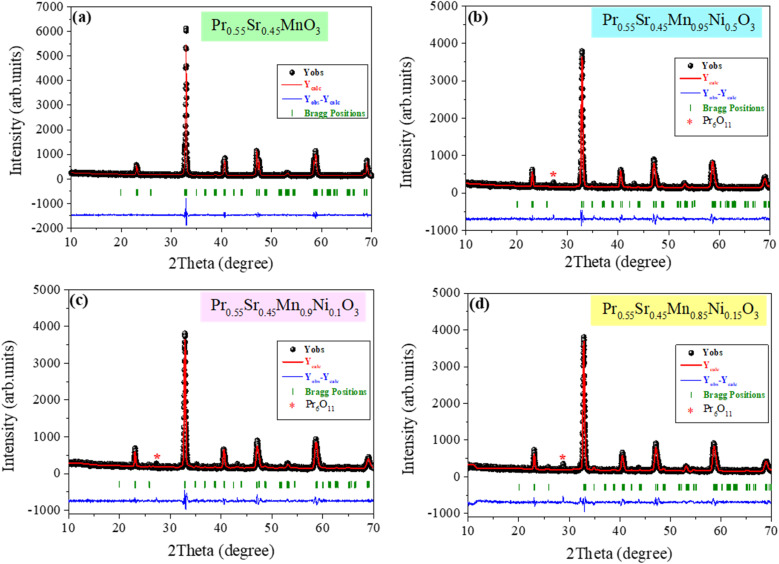
X-ray powder diffraction patterns and refinement at ambient temperature for the Pr_0.55_Sr_0.45_Mn_1−*x*_Ni_*x*_O_3_ samples: ((a) *x* = 0, (b) *x* = 0.05, (c) *x* = 0.1 and (d) *x* = 0.15). The differences in the calculated and observed intensities along the Bragg positions are provided (vertical bars in green).

Fig. 1(a–d) display the X-ray diffraction patterns of Pr_0.55_Sr_0.45_Mn_1−*x*_Ni_*x*_O_3_ at room temperature with *x* = 0.0, 0.05, 0.1, and 0.15, respectively. The X-ray diffraction XRD analysis indicates that the Pr_0.55_Sr_0.45_Mn_1−*x*_Ni_*x*_O_3_ compounds have an orthorhombic structure with the *Pnma* space group. The absence of peaks corresponding to a secondary phase with a very small amount of Pr_6_O_11_ for *x* > 0 values confirmed the quality of the developed compounds and their single phase. We used the FullProf software to perform Rietveld refinement^50–52^ with the objective to better understand the structural properties of the orthorhombic phase. The standard agreement indicators *R*_wp_, *R*_p_, and *R*_F_, and the goodness of fit *χ*^2^ were used to evaluate the reliability and quality of the Rietveld refinement. A statistically reliable measure of the overall fit quality, the weighted profile *R*-factor, *R*_wp_, quantifies the weighted difference between the calculated and observed diffraction intensities, however, the profile *R*-factor (*R*_p_) offers an unweighted indicator of the total difference between the calculated and experimental patterns. The structure *R*-factor (*R*_F_) measures how well the calculated and observed structure factor amplitudes agree, underlining the precision of the underlying structural model. The refinement quality is normalized with respect to the expected statistical noise using the goodness of fit parameter (*χ*^2^), where 
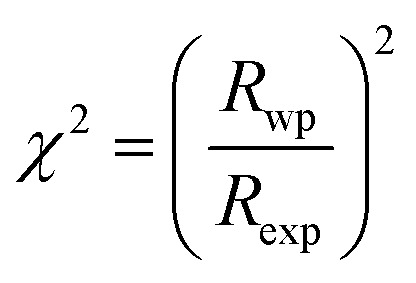
 is calculated using the expected residual, *R*_exp_, which is the statistically lowest value of *R*_wp_ based on counting statistics. The low values of *R*_wp_, *R*_p_, and *R*_F_ in this investigation, along with *χ*^2^ values near unity, show a successful and dependable refinement that is in good agreement with the literature.^53^ Furthermore, the average crystallite size (*D*) was calculated using the main peak in the XRD pattern using the Debye–Scherrer formula:1
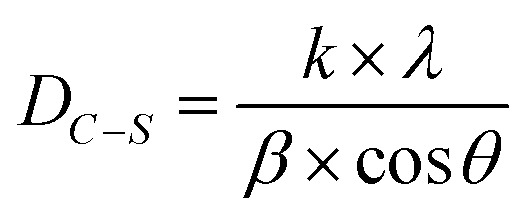
where *k* indicates a dimensionless constant (*k* = 0.9), *β* defines the peak half-height width, and *θ* represents the peak Bragg angle.

However, the X-ray density, *ρ*_X-ray_, was determined using the following formula for all samples:2
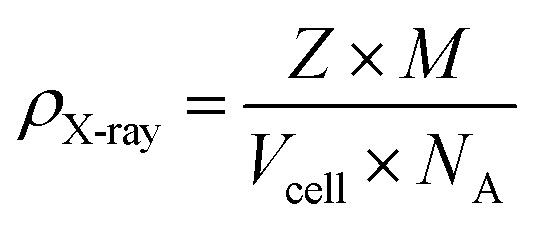
where *V*_cell_ is the unit cell volume (cm^3^), *M* is the molar mass of the sample (in g mol^−1^), *N*_A_ = 6.022 × 10^23^ mol^−1^ is the Avogadro number, and *Z* = 4 is the number of formula units per unit cell for the orthorhombic perovskite structure with the *Pnma* space group.

Thus, the compound bulk density,*ρ*_exp_, was determined using the following formula:3
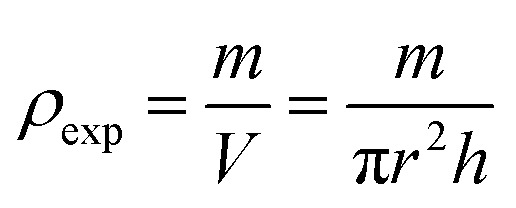
where *h*, *r*, and *m* correspond to the thickness, radius, and mass of the pellet, respectively.^54^Table 1 displays the values of various structural parameters, lattice parameters (*a*, *b*, and *c*), unit volume, bond lengths, bond angles, goodness of fit, *χ*^2^, *ρ*_X-ray_, *ρ*_exp_, *etc.*, using Rietveld refinement.

**Table 1:** Structural properties of the Pr_0.55_Sr_0.45_Mn_1−*x*_Ni_*x*_O_3_ compounds, where *x* = 0.0, 0.05, 0.1, and 0.15, at ambient temperature, as determined by the Rietveld refinement method

*x*	0	0.05	0.10	0.15
*a* (Å)	5.4409	5.4529(6)	5.46048	5.4586(3)
*b* (Å)	7.6558	7.7233(7)	7.67110	7.7276(5)
*c* (Å)	5.4807	5.3951(12)	5.46381	5.4011(6)
*V* (Å^3^)	228.296(0.018)	227.209(0.058)	228.898(0.000)	227.827(0.032)
Bragg *R*-factor	3.79	4.96	4.53	5.32
Rf-factor	7.52	7.04	4.85	7.41
(<*d*_Mn1–O_2__>) (Å)	2.06232	1.68235	1.86293	1.57848
(Mn1)-(O_2_)-(Mn1)	173.244	161.561	152.568	173.937
(<d_Mn2–O_2__>) (Å)	1.80577	2.20233	2.1123	2.26627
(Mn2)-(O_2_)-(Mn2)	173.244	161.561	152.568	173.937
(<d_Mn1–O_1__>) (Å)	1.94945	1.93466	1.93455	1.97244
(Mn1)-(O_1_)-(Mn1)	158.099	172.780	164.897	156.727
*R*_e_	16.4	17.4	18.0	14.5
*R*_p_ (%)	30.5	33.5	37.9	27.4
*R*_wp_ (%)	20.5	23.4	24.5	21.3
*χ* ^2^ (%)	1.56	1.82	1.85	2.15
*D*_c–s_ (nm)	78	53	47	46
*ρ*_X-ray_ (g cm^−3^)	6.40	6.43	6.39	6.43
*ρ*_exp_ (g cm^−3^)	6.28	6.10	6.19	6.02

To fully understand the structural and functional properties present in our Pr_0.55_Sr_0.45_Mn_1−*x*_Ni_*x*_O_3_ series of compounds where *x* = 0.0, 0.05, 0.1, and 0.15, careful analysis of their crystallographic parameters is necessary, particularly their inter-octahedral <*d*_Mn–O_> bond and the <*θ*_Mn–O–Mn_> angles.

The <*d*_Mn–O_> interatomic distances directly reveal the size and degree of deformation of the MnO_6_ octahedra. When Ni replaces Mn in the Pr_0.55_Sr_0.45_Mn_1−*x*_Ni_*x*_O_3_ structure, the average oxidation state of the manganese ions is drastically altered, leading to the introduction of certain structural stresses. The resulting changes in the <d_Mn–O_> bond lengths also have a significant impact on the Jahn–Teller effect, which is often associated with the presence of Mn^3+^ cations. The electrical asymmetry of these ions causes the octahedra to compress or elongate, which is essential for creating the local structural environment.

At the same time, the <*θ*_Mn–O–Mn_> bond angles control the connection and coupling efficacy between adjacent MnO_6_ octahedra. The degree of nickel (Ni) substitution directly affects the amount of octahedral tilt. This tilt is very important because it plays a critical role in mediating the double exchange (DE) process in Mn^3+^–O–Mn^4+^ that generates the observed ferromagnetism in these manganites.

Specifically, when the <*θ*_Mn–O–Mn_> angle approaches the ideal 180°, the orbital overlap is maximized, strengthening the magnetic interactions and increasing the charge carrier mobility.

In summary, understanding the magnetic characteristics observed in Pr_0.55_Sr_0.45_Mn_1−*x*_Ni_*x*_O_3_ requires analyzing the simultaneous changes in the <*d*_Mn–O_> distances regulated by the Jahn–Teller effect and the substitution as well as the <*θ*_Mn–O–Mn_ > angles that influence the efficacy of the double-exchange mechanism.

### 3.2 Magnetic properties

**Fig. 2:**
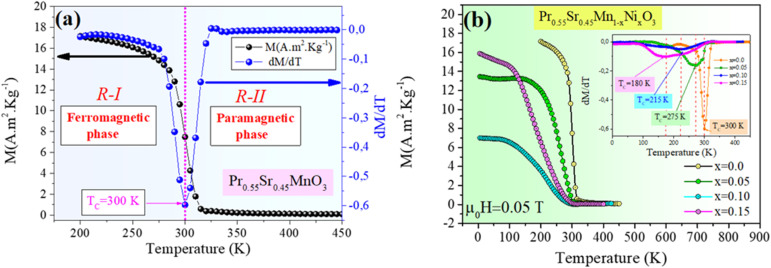
(a) Temperature dependence of magnetization measured at a magnetic field of *µ*_0_*H* = 0.05 T for the Pr_0.55_Sr_0.45_MnO_3_ sample and dM/dT *versus* temperature curves. (b) Magnetization *vs.* temperature plots for the Pr_0.55_Sr_0.45_Mn_1−*x*_Ni_*x*_O_3_ (*x* = 0, *x* = 0.05, *x* = 0.1 and *x* = 0.15) compounds measured at *µ*_0_*H* = 0.05 T. Inset: dM/dT against temperature graphs for the perovskite compounds.

Fig. 2(a) shows the plot of magnetization as a function of temperature under a weak applied field of *µ*_0_*H* = 0.05 T, with the d*M*/d*T* curve used to determine *T*_C_ with the minimum of the parent sample curve, clearly demonstrating the paramagnetic to ferromagnetic transition (PM–FM) transition.

The temperature dependence of the magnetization, *M*(*T*), under an applied magnetic field of *µ*_0_*H* = 0.05 T for all our samples is displayed in Fig. 2(b). The magnetization *vs.* temperature *M*(*T*) curves for the Pr_0.55_Sr_0.45_Mn_1−*x*_Ni_*x*_O_3_ series, where *x* = 0.0, 0.05, 0.1, and 0.15, offer important insights regarding their magnetic behavior. As the temperature decreases, these compounds exhibit a transition from the PM to FM state. The temperature at which a material transitions from a paramagnetic to a ferromagnetic state is indicated by the key value known as the Curie temperature, *T*_C_. The minimum value of the d*M*/d*T vs. T* curves was used to calculate *T*_C_ (see the inset of Fig. 2(b)).

As the percentage of doping Ni increases, the *T*_C_ values for the Pr_0.55_Sr_0.45_Mn_1−*x*_Ni_*x*_O_3_ compounds are shown to decline: *T*_C_ (K) = 300 K, 275 K, 215 K, and 180 K for *x* = 0.0, 0.05, 0.10, and 0.15, respectively. The observed behavior suggests that substituting Ni for Mn in the lattice lowers the ferromagnetic interactions, which reduces the *T*_C_. This has been confirmed in the literature by the work of A. K. Saw and collaborators^55^ and other studies.^56^

In addition, the magnetism in the FM region also decreases when Ni is substituted in the Pr_0.55_Sr_0.45_MnO_3_-doped manganites. The decrease in the Mn^3+^/Mn^4+^ ratio provides a qualitative justification for the decrease in *T*_C_ and magnetism with an increasing nickel concentration. By decreasing the Mn^3+^/Mn^4+^ couples that result in DE ferromagnetism and adding a tiny quantity of Mn^3+^/Mn^3+^ and Mn^4+^/Mn^4+^ couples, this action raises the SE antiferromagnetic state.^57^

**Fig. 3:**
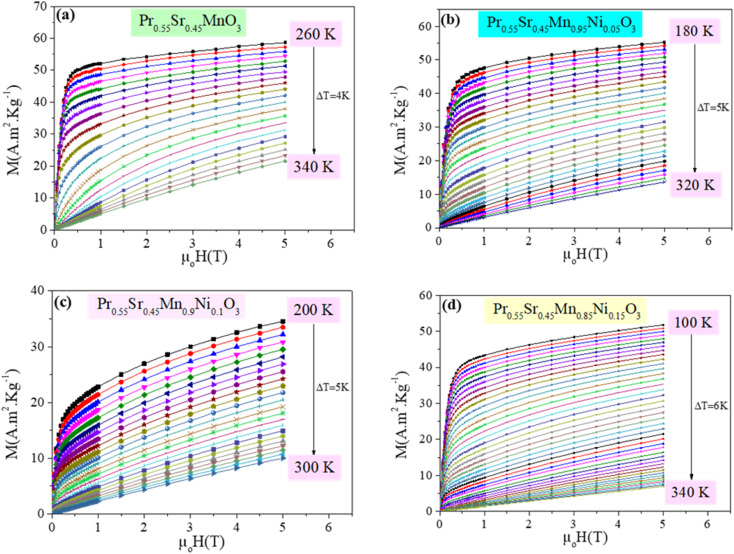
Isothermal magnetization *M*(*H*) curves at different temperatures (*T*) around the Curie temperature (*T*_c_) for the Pr_0.55_Sr_0.45_Mn_1−*x*_Ni_*x*_O_3_ perovskite samples. ((a) *x* = 0, (b) *x* = 0.05, (c) *x* = 0.1 and (d) *x* = 0.15).

Over a broad temperature range of 100–340 K, the magnetization isotherms *M*(*H*) recorded for all the compounds in a magnetic field of *µ*_0_*H* equal to 5 T show that below *T*_C_, the magnetization increases significantly in weak applied fields until approaching saturation for an applied field of *µ*_0_*H* = 1 T. For *x* = 0.0, 0.05, 0.1, and 0.15, Fig. 3(a)–(d) show the typical magnetization isotherm form. The saturation magnetization increases with a decrease in temperature. This result confirms the exclusively ferromagnetic nature of these materials at low temperatures. The magnetization *versus* magnetic field *M*(*H*) measurements provide important information about the magnetic properties and phase transitions of manganites.

These measurements indicate the nature of the magnetic ordering and the transitions between different magnetic phases. In this regard, a second-order FM to PM phase transition can be observed in the *M*(*H*) curves for Pr_0.55_Sr_0.45_Mn_1−*x*_Ni_*x*_O_3_ (0.0 ≤ *x* ≤ 0.15), where the *T*_C_ decreases with an increase in the Ni-doping concentration. Similarly, *M*(*H*) measurements reveal a second-order PM–FM transition in the study by A. Dhahri and collaborators for Pr_0.76_Sr_0.33_Mn_1−*x*_M_*x*_O_3_ (0.0 < *x* ≤ 0.09).^58^ Understanding magnetic interactions and materials with potential applications in magnetic refrigeration and other technologies is also important.

### 3.3 Critical behavior

Determining the critical behavior at the critical point is made possible by the examination of the Arrott curve. For a system that follows the theory of the average-field MFT, the magnetic equation of state is as follows:

4 *µ*_0_*H*/*M* = *A* + BM^2^

A comprehensive investigation of *M*(H) isotherms indicates the order of the magnetic phase transition. In this way, based on the Arrott–Noakes equation of state,^59^ the MFT can be expanded to be a so-called modified Arrott plot expression to describe the second-order phase transition, as follows:

5 (*µ*_0_*H*/*M*)^1/*γ*^ = *a*(*T* − *T*_C_)/*T*_C_ + 4b M^1/*β*^

**Fig. 4:**
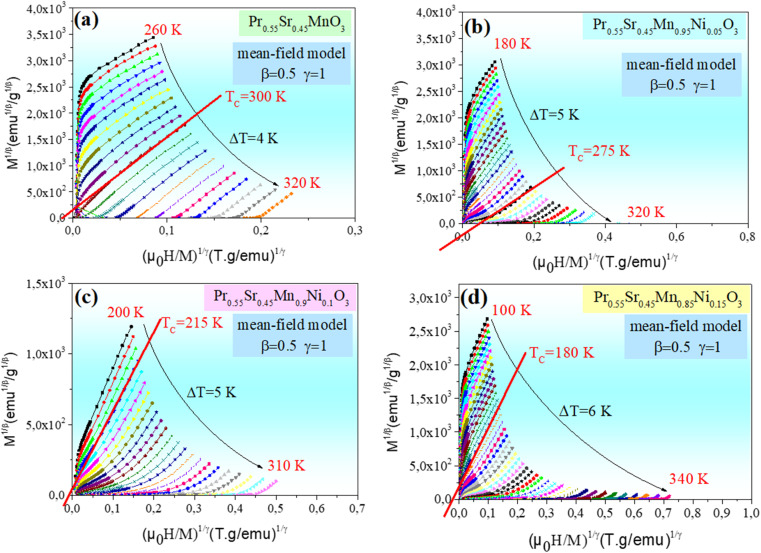
The Arrott plots (M^1/*β*^*vs.*(*µ*_0_*H*/*M*)^1/*γ*^) in main-field model with (*β* = 0.5 and *γ* = 1) around *T*_c_, of Pr_0.55_Sr_0.45_Mn_1−*x*_Ni_*x*_O_3_ ((a) *x* = 0, (b) *x* = 0.05, (c) *x* = 0.1 and (d) *x* = 0.15) samples.

The Arrott plots (*M*^1/*β*^*vs.* (*µ*_0_*H*/*M*)^1/*γ*^), which are displayed in Fig. 4(a)–(d), were derived from the *M*(*H*) isotherms near the *T*_C_. According to the Banerjee criterion,^60^ a negative or positive slope in the Arrott curve indicates a first-order or second-order magnetic phase transition, respectively.^61^

The *M*^2^ against *µ*_0_*H*/*M* graphs for Pr_0.55_Sr_0.45_Mn_1−*x*_Ni_*x*_O_3_ show a second-order FM to PM phase transition, with a positive slope in every instance in the whole *M*^2^ range. The values of exponents *β* and *γ* are taken to be 0.5 and 1, respectively, as predicted by mean field theory. For all the samples, the *T*_C_ found using the Arrott plots match those found in the low-field magnetization curves, *M*(*T*).

**Fig. 5:**
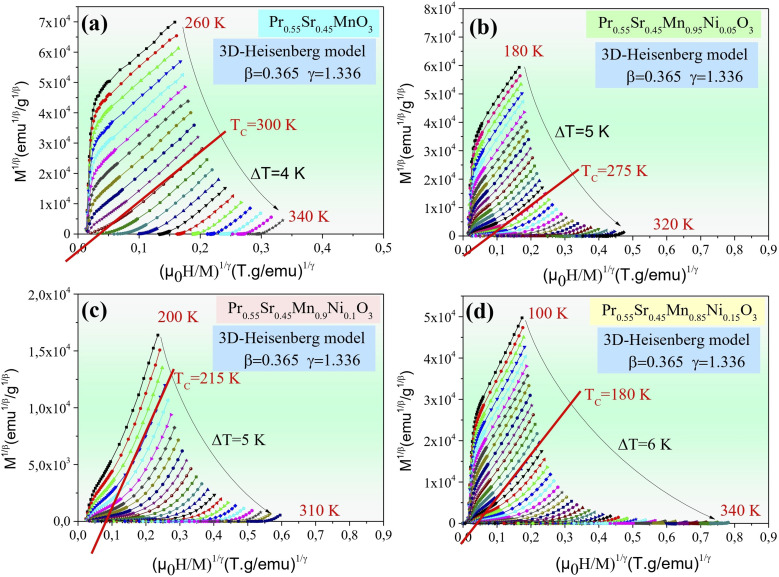
Isotherms of (*M*^1/*β*^*vs.*(*µ*_0_*H*/*M*)^1/*γ*^) in 3D-Heisemberg model with (*β* = 0.365 and *γ* = 1.336) around *T*_c_ for Pr_0.55_Sr_0.45_Mn_1−*x*_Ni_*x*_O_3_ ((a) *x* = 0, (b) *x* = 0.05, (c) *x* = 0.1 and (d) *x* = 0.15) perovskite samples.

**Fig. 6:**
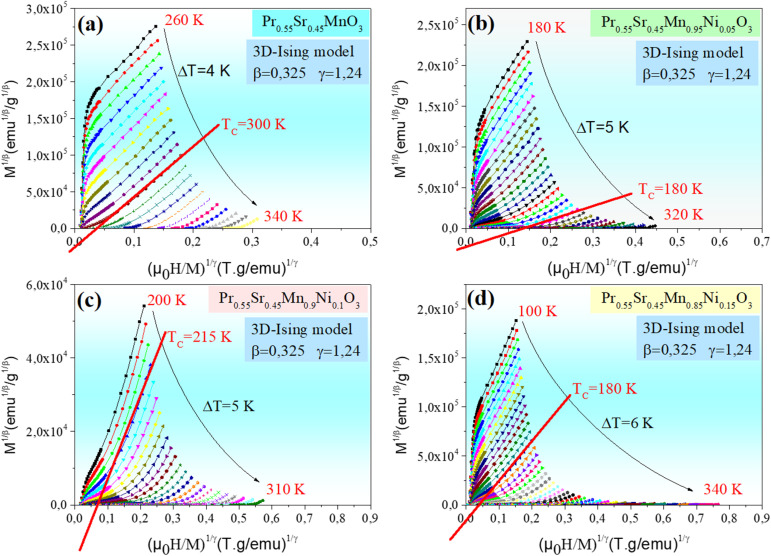
Isotherms of (*M*^1/*β*^*vs.*(*µ*_0_*H*/*M*)^1/*γ*^) in 3D-Ising model with (*β* = 0.325 and *γ* = 1.24) around *T*_c_ for Pr_0.55_Sr_0.45_Mn_1−*x*_Ni_*x*_O_3_ ((a) *x* = 0, (b) *x* = 0.05, (c) *x* = 0.1 and (d) *x* = 0.15) perovskite samples.

**Fig. 7:**
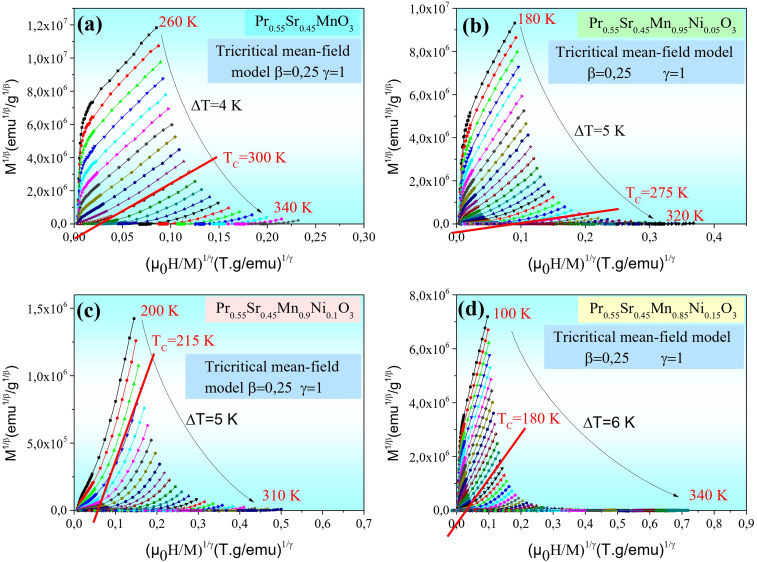
Isotherms of (*M*^1/*β*^*vs.*(*µ*_0_*H*/*M*)^1/*γ*^) in tritical mean field model with (*β* = 0.25 and *γ* = 1) around *T*_c_ for Pr_0.55_Sr_0.45_Mn_1−*x*_Ni_*x*_O_3_ ((a)*x* = 0, (b) *x* = 0.05, (c) *x* = 0.1 and (d) *x* = 0.15) perovskite samples.

Fig. 4–7 show the modified Arrott plots, MAP, at different temperatures for all the Pr_0.55_Sr_0.45_Mn_1−*x*_Ni_*x*_O_3_ compounds, where *x* = 0.00, 0.05, 0.1, and 0.15, using 4 theoretical models: mean field model (*β* = 0.5, *γ* = 1) in Fig. 4(a–d), 3D Heisenberg model (*β* = 0.365, *γ* = 1.336 in Fig. 5(a–d), 3D-Ising model (*β* = 0.325, *γ* = 1.24) in Fig. 6(a–d), and tricritical mean-field model (*β* = 0.25, *γ* = 1) in Fig. 7(a–d).

It is clear in the modified Arrott plots at lower field that these isotherms are curved because they are averaged across domains with varying magnetization. However, all the isotherms are nearly parallel to one another in high fields, and the mean field model is more accurate if the intercept of these lines on the (*µ*_0_*H*/*M*)^1/*γ*^ axis is negative or positive below or above *T*_C_ and the line *M*^1/*β*^*versus* (*µ*_0_*H*/*M*)^1/*γ*^ at *T*_C_ passes through the origin (see Fig. 4–7).

As observed, the mean field model is the ideal one for studying the critical behavior of the Pr_0.55_Sr_0.45_Mn_1−*x*_Ni_*x*_O_3_ compounds, where 0 ≤ *x* ≤ 0.15. In particular, it is clear from Fig. 4 that all the isotherms are almost parallel and linear in the high-field region, and the intercept of these lines on the (*µ*_0_*H*/*M*)^1/*γ*^ axis is a positive slope above *T*_C_ and the line of (*M*^1/*β*^*vs.* (*µ*_0_*H*/*M*)^1/*γ*^) at *T*_C_ = 300, 275, 215 and 180 K for Pr_0.55_Sr_0.45_Mn_1−*x*_Ni_*x*_O_3_, where *x* = 0.00, 0.05, 0.1, and 0.15, respectively, almost passes through the origin.

According to the Banerjee criterion, all the mean field model plots for our samples show positive slopes, which support their ferromagnetic–paramagnetic transition second-order nature. The improved agreement with the mean field model indicates that in the composition range under study, long-range magnetic interactions predominate in the magnetic behavior of Pr_0.55_Sr_0.45_Mn_1−*x*_Ni_*x*_O_3_.

**Fig. 8:**
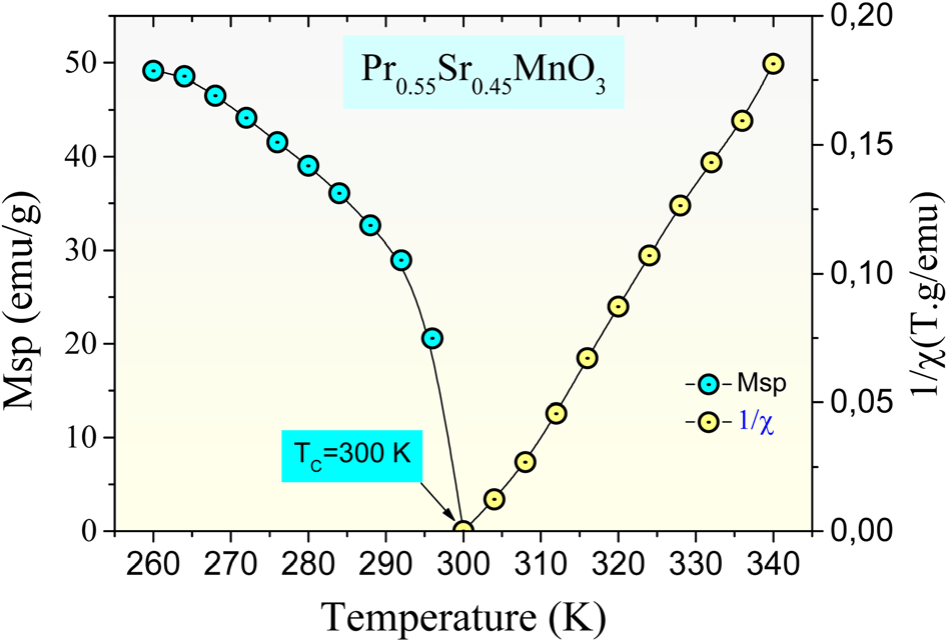
Spontaneous magnetization, *M*_sp_(*T*) (left-axis) and inverse susceptibility 1/*χ* (right-axis) as a function of temperature for the Pr_0.55_Sr_0.45_Mn_1−*x*_Ni_*x*_O_3_ ((a) *x* = 0, (b) *x* = 0.05, (c) *x* = 0.1 and (d) *x* = 0.15) perovskite samples.

For the Pr_0.55_Sr_0.45_MnO_3_ parent sample, Fig. 8 displays the temperature dependence of the inverse susceptibility (1/*χ*), and the spontaneous magnetization (*M*_sp_) is derived from the *M*(*H*) curves. In the paramagnetic phase (*T* > *T*_C_), this sample exhibits linear inverse susceptibility behavior, *χ* = *C*/(*T*−*θ*_P_), where *θ*_P_ is the Curie constant and *C* is the Curie–Weiss temperature. For 0% Ni-doped, the *θ*_P_ value is determined to be 303 K. The presence of an FM exchange interaction between the closest neighbors is indicated by the positive value of *θ*_P_. The resultant value is marginally above the Curie temperature. We may conclude that this discrepancy varies depending on the material and is associated with the existence of short-range ordered somewhat above the Curie temperature, *T*_C_, which is associated with the existence of magnetic inhomogeneity.^62^ For the parent compound, the critical exponent *β* is determined to be 0.362, indicating that all our samples exhibit ferromagnetic activity at low temperatures. The ferromagnetic condition described for manganites^63^ is in good agreement with this value.

### 3.4 Magnetocaloric measurements

According to thermodynamic theory, the magnetic entropy change caused by a variation in the magnetic field from 0 to *µ*_0_*H*_max_ is as follows:^64^6
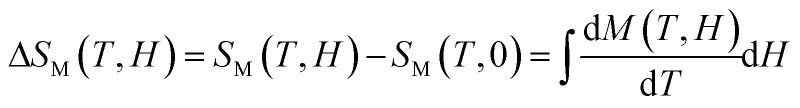


Applying Maxwell's relation7
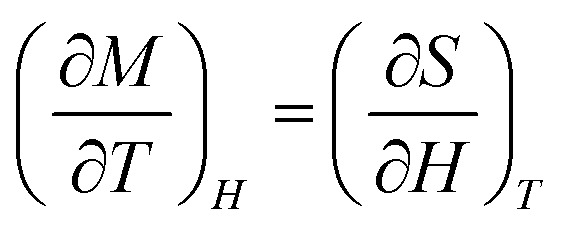


The following expression can be obtained:8
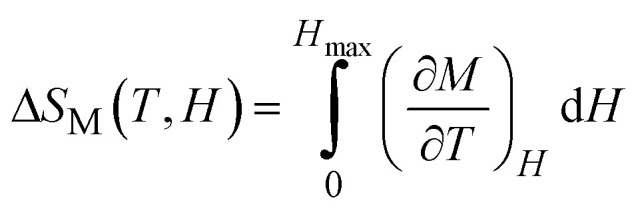


**Fig. 9:**
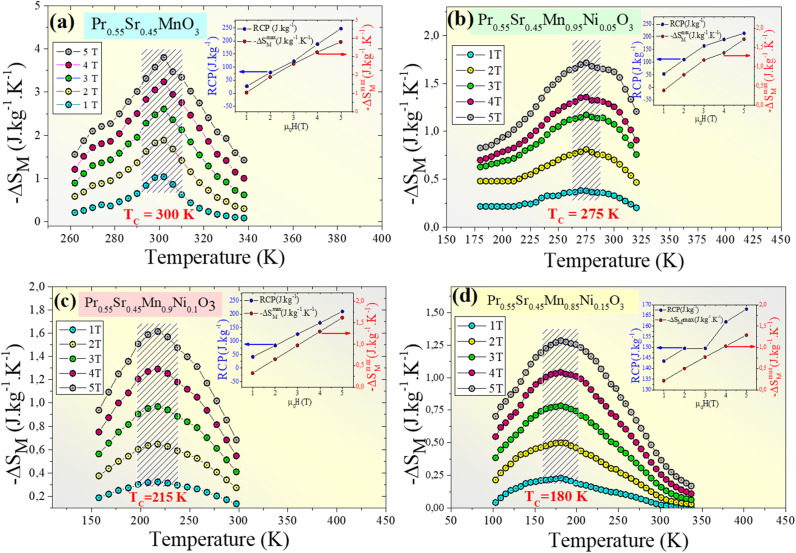
Magnetic entropy change (−Δ*S*_M_) *versus T* around the Curie temperature (*T*_c_) for the Pr_0.55_Sr_0.45_Mn_1−*x*_Ni_*x*_O_3_ ((a) *x* = 0, (b) *x* = 0.05, (c) *x* = 0.1 and (d) *x* = 0.15) compounds. Insets of Fig. 9. Relation between the maximum entropy change −Δ*S*^max^_M_) and relative cooling power (RCP) at various applied magnetic fields (*µ*_0_*H*) for the Pr_0.55_Sr_0.45_Mn_1−*x*_Ni_*x*_O_3_ compounds.

According to eqn (8), the magnetic transition phase is when the magnetic entropy change is at its maximum. Using isothermal magnetization data in small discrete fields and temperature intervals, the magnetic entropy change can be accurately approximated using the following numerical formula:^65^9
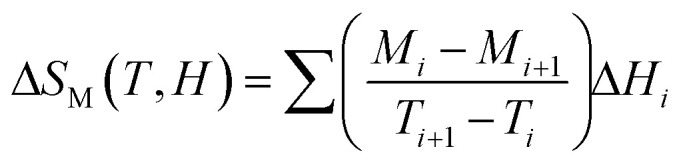
where *M*_*i*_ and *M*_*i*+1_ are the experimental magnetization values obtained at temperatures *T*_*i*_ and *T*_*i*+1_, respectively, in applied magnetic field *H*_*i*_. Fig. 9(a–d) plot the magnetic entropy change, |Δ*S*_M_|, of the Pr_0.55_Sr_0.45_Mn_1−*x*_Ni_*x*_O_3_ samples as a function of temperature under different magnetic applied field variations. It increases and peaks in the vicinity of the magnetic-transition temperature. For all the compounds, −Δ*S*_M_ shows a wide positive peak at around *T*_C_. The maximum values of the magnetic entropy change,|Δ*S*^max^_M_|, are 3.8 J kg^−1^ K^−1^, 1.71 J kg^−1^ K^−1^, 1.62 J kg^−1^ K^−1^ and 1.29 J kg^−1^ K^−1^ in a magnetic field change of Δ*µ*_0_*H* = 5 T for 0%, 5%, 10% and 15%, respectively (Table 2).

**Table 2:** Summary of the −Δ*S*^max^_M_, *T*_c_ and RCP values for Pr_0.55_Sr_0.45_Mn_1−*x*_Ni_*x*_O_3_ where *x* = 0.0, 0.05, 0.1, and 0.15

*x*	0	0.05	0.10	0.15
*T*_C_ (K)	300	275	215	180
−Δ*S*^max^_M_(J kg^−1^ k^−1^)	3.80	1.71	1.62	1.29
*δT*_FWHM_	65	125	130	168
RCP (J kg^−1^)	247	216	211	215.94

As seen in Fig. 11, the noted decrease in the peak of magnetic entropy changes, −Δ*S*^max^_M_, with increasing Ni-doping is explained by the modification of the Mn^3+^/Mn^4+^ structure, which impacts the strength of the double-exchange interaction responsible for FM ordering. When Mn^3+^ is replaced by Ni^2+^, the Mn^3+^–O–Mn^4+^ pairs decrease. The decline in the long-range FM coupling and the decrease in overall magnetization near the *T*_C_ directly impact the magnetocaloric effect, which manifests as a decrease in −Δ*S*^max^_M_ with an increasing nickel doping percentage. This interpretation is consistent with subsequent studies on Ni-doped perovskite-type manganites, where a decrease in the Mn^3+^–O–Mn^4+^ double-exchange interaction reduces −Δ*S*^max^_M_. Furthermore, the Ni substitution effect provides a clear microscopic mechanism explaining the direct decrease in the magnetocaloric phenomenon when increasing the percentage of nickel.^66^

The relative cooling power (RCP)^67–69^ is calculated as follows:

10 RCP = −Δ*S*^max^_M_(*T*,*H*) × *δT*_FWHM_

where *δT*_FWHM_ is the full-width at half-maximum of −Δ*S*_M_ against *T*_C_.^70–72^ For our compounds, the RCP values are 247 J kg^−1^, 216 J kg^−1^, 211 J kg^−1^ and 215.94 J kg^−1^ at *µ*_0_*H* = 5 T for 0%, 5%, 10% and 15%, respectively. Due to its high RCP value, the composition with 0% nickel doping appears to be the most promising for magnetic refrigeration applications. The entropy change and temperature range must be matched for cooling cycles to be successful. The inset of Fig. 9(a–d) shows the delta |Δ*S*^max^_M_| values obtained, and the RCP values calculated using eqn (7) are plotted *versus µ*_0_*H*.

It was noticeable that at *T*_C_, the RCP and |Δ*S*^max^_M_| are both proportional to *µ*_0_*H*. The pattern of RCP with *µ*_0_*H* indicates that it is a field-dependent variable. Materials with high *δT*_FWHM_ and RCP values can function over a wide temperature range and have a significant cooling capacity. Table 3 shows that compared with gadolinium (Gd), our studied compounds have a significant value of −Δ*S*^max^_M_, a large RCP, and a tunable *T*_C_ from room temperature to below room temperature, making them suitable solid-state refrigerant materials. The RCP value of our 0% nickel sample of 247 J kg^−1^ is 56% of the RCP value of Gd, which is the industry standard for refrigeration.^73^ Thus, these materials are well-suited for magnetic refrigeration.^74,75^

**Table 3:** Collected data for Curie temperature (*T*_c_), −Δ*S*^max^_M_, and RCP for our studied compounds and the reference material Gd

*x*	*T*_c_ (K)	Δ*H* (*T*)	−Δ*S*^max^_M_(J kg^−1^ k^−1^)	RCP (J kg^−1^)	Refs
*x* = 0	300	5	3.8	247	This work
*x* = 0.05	275	5	1.71	216	This work
*x* = 0.1	215	5	1.62	211	This work
*x* = 0.15	180	5	1.29	215.94	This work
Gd	294	5	10.2	410	9

Several methods for determining the order of magnetic phase transitions have been proposed in the literature.^76,77^ One of these methods is to analyze the field dependency of the MCE of the samples using the relation Δ*S*_M_(*H*,*T*) ≈ *aH*^*n*^, where ‘*a*’ is a constant and ‘*n*’ is an exponent associated with magnetic order.^78–80^ By calculating the exponent values at particular temperatures, significant information can be obtained on the specific type of magnetic phase transition in the materials under investigation. The exponent *n* at a specific temperature and magnetic field can be found using the logarithmic derivative of the experimental data Δ*S*_M_(*H*,*T*):^81^11
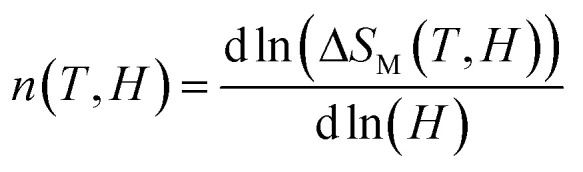


At high temperatures in the paramagnetic phase (above the *T*_C_), the *n* values approach *n* = 2 according to the Curie–Weiss rule.^82^ At temperatures much lower than the transition point, *n* usually tends to be *n* = 1 in the ferromagnetic phase. Within a critical temperature range around the Curie temperature, the value of *n* varies according to the type of phase transition (first or second-order) of the material.

**Fig. 10:**
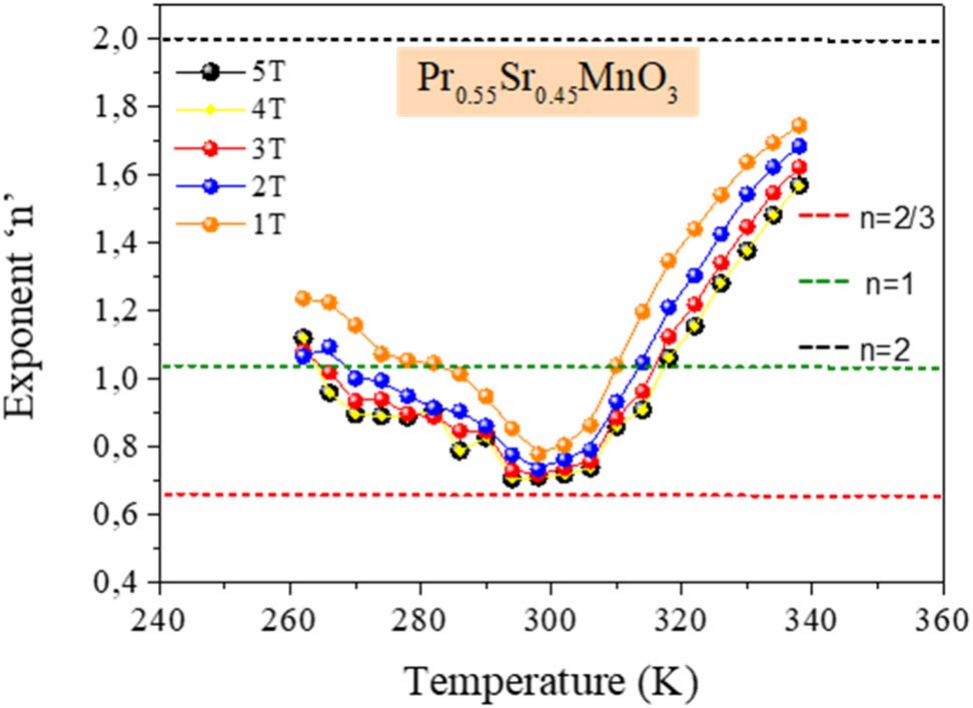
Temperature dependence of the exponent *n* (*T*), for various applied magnetic field values Δ*µ*_0_*H* for Pr_0.55_Sr_0.45_Mn_1−*x*_Ni_*x*_O_3_.

A quantitative requirement of *n* > 2 near the Curie temperature *T* = *T*_C_ has been shown in earlier research to be consistent with a first-order magnetic phase transition of a material. Additionally, it has been shown that this criterion can be effectively used to determine the type of magnetic phase transition in a range of magnetocaloric materials.^83^ The temperature dependence of exponent *n* for the Pr_0.55_Sr_0.45_MnO_3_ compound under different magnetic fields *n*(*T*) is shown in Fig. 10. At temperatures much lower than *T*_C_ (ferromagnetic zone), the sample exponent *n* tends to approach 1. For temperatures higher than *T*_C_ (paramagnetic zone), exponent *n* goes to 2. Usually, a second-order phase transition explains this behavior for exponent *n*. A decrease in *n* is observed as the temperature approaches the transition temperature, reaching its lowest value at *T*_C_. Similar behavior has been documented for other magnetic materials with first- and second-order transitions.^84–87^

**Fig. 11:**
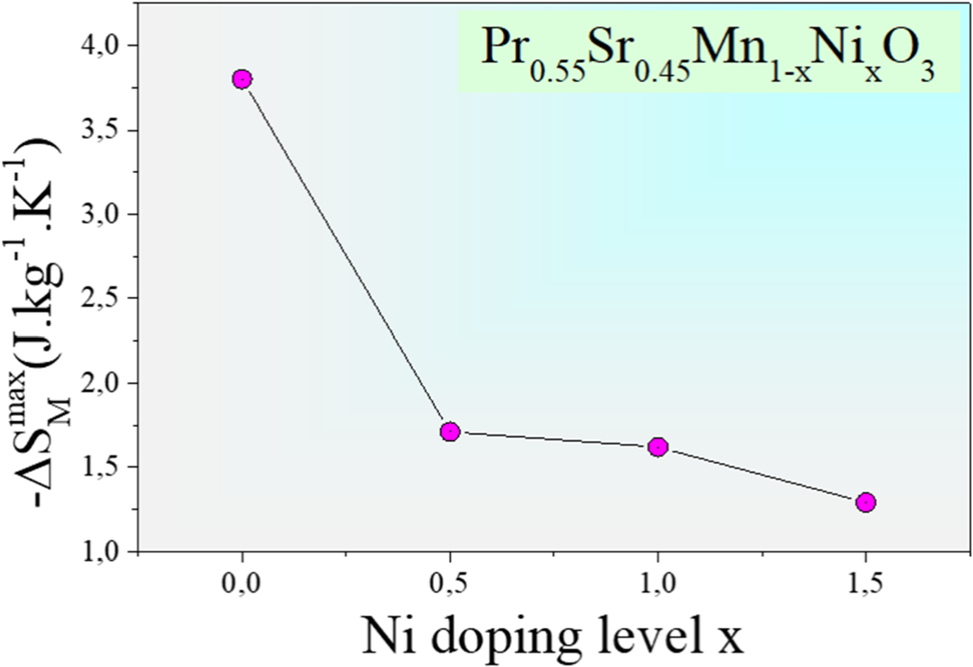
Effect of nickel doping on the peak magnetic entropy change −Δ*S*^max^_M_ for the Pr_0.55_Sr_0.45_Mn_1−*x*_Ni_*x*_O_3_ (0.0 ≤ *x* ≤ 0.15 compounds.

## 4. Conclusions

In conclusion, we carried out detailed studies, such as structural, magnetic, and magnetocaloric characterization of Pr_0.55_Sr_0.45_Mn_1−*x*_Ni_*x*_O_3_ manganite compounds, where *x* = 0.0, 0.05, 0.1, and 0.15, which were successfully elaborated utilizing a solid-state reaction. The X-ray diffraction study confirmed the generation of a single phase over the compositional range, and the crystalline structure was determined to be orthorhombic, corresponding to the *Pnma* space group.

Magnetization measurements as a function of applied field and temperature consistently indicated a magnetic phase transition from the ferromagnetic to paramagnetic state for all our compounds. Remarkably, this transition was found to be of the second order.

It was found that gradually substituting nickel for manganese may effectively control the Curie temperature, *T*_C_, which decreased from 300 K to 180 K with the studied substitution term. This dependence demonstrates the critical influence of the Ni^2+^ ion on the magnetic exchange processes within the crystal lattice. Using the isothermal magnetization data and the Maxwell thermodynamic laws, the magnetocaloric effect was thoroughly examined. The results of a magnetic field change of 5 T are highly promising. The maximum magnetic entropy change, −Δ*S*^max^_M_, for the *x* = 0 composition was found to peak at 3.8 J kg^−1^ K^−1^. More importantly, at 247 J kg^−1^, the Pr_0.55_Sr_0.45_MnO_3_ sample had the highest relative cooling power (RCP) value. This result sets the Pr_0.55_Sr_0.45_MnO_3_ material in a promising category for magnetic refrigeration applications, given that its RCP value is around 56% of that of pure gadolinium, an established industry measurement. In conclusion, the Pr_0.55_Sr_0.45_Mn_1−*x*_Ni_*x*_O_3_ family of manganites is an appealing class of magnetocaloric materials for the development of efficient and environmentally friendly magnetic cooling devices, particularly because their operating temperature can be adjusted based on the nickel concentration.

## Conflicts of interest

There are no conflicts to declare.

## Data Availability

The data that support the findings of this study are available from the corresponding author upon reasonable request.
